# Production of dumbbell probe through hairpin cleavage-ligation and increasing RCA sensitivity and specificity by circle to circle amplification

**DOI:** 10.1038/srep29229

**Published:** 2016-07-07

**Authors:** Hua Wei, Suming Tang, Tianyu Hu, Guojie Zhao, Yifu Guan

**Affiliations:** 1 Animal Science and Veterinary Medicine College, Shenyang Agricultural University, #120 Dongling Road, Shenyang, Liaoning, 110866, China; 2 Department of Biochemistry and Molecular Biology, China Medical University, #77 Puhe Road, Shenyang, Liaoning, 110122, China

## Abstract

Dumbbell probe (DP) attracts increasing interests in rolling circle amplification (RCA). A universal DP production method through cleavage-ligation of hairpin was proposed and optimized. The production is characterized by restriction endonuclease (RE)-induced cleavage ends ligation. It has the advantage of phosphorylation-free, splint-free and purification-free. To optimize designing, we found that the position of RE cleavage sequence in the stem and the primer position in the loop affected the formation and amplification of DP obviously. Both sticky and blunt ends cleaved by RE produce DP efficiently. Moreover, we introduced this DP into circle to circle (C2C) RCA based on the same cleavage-ligation principle, and acquired high sensitivity. By combining a two-ligation design and the C2C strategy, specificity for detecting let-7 family members was increased extremely. Furthermore, coreaction of different steps facilitated convenient formation and amplification process of DP.

Nucleic acids detection is one of the most promising molecular diagnosing technologies. Its application can range from the traditional single nucleotide polymorphism (SNP) detection to the increasingly discovered microRNA detection^1^,^2^,^3^. Various detection technologies are available, such as polymerase chain reaction (PCR) and its derivatives. Different from these thermal recycle amplification methods, isothermal amplification methods have attracted more and more interests. The main advantage of this kind of technology is the constant reaction temperature independent on a thermal recycler. Rolling circle amplification (RCA) is an attracting isothermal amplification method with the advantage of sensitivity and simplicity. It has been exploited to various methods for detecting biosamples such as cells^4^, DNA^5^, RNA^6^, proteins^7^ and small molecules^8^. In addition, it is also a powerful tool for enzymatic catalysis assay, such as asymmetric cleavage of restriction endonuclease (RE)^9^, methylation assay^10^, topoisomerase activity^11^ and polynucleotide kinase catalysis^12^. RCA is mainly characterized by a circular single-stranded DNA (ssDNA) template. DNA polymerases bearing strand-displacing activity, such as phi29 DNA polymerase, can extend primer many rounds along circular template, and release large macromolecule ssDNA products with tandem sequences complementary to the template. Thus, a key point for all RCA-based methods is to prepare a circular DNA template.

Though RCA amplification and detection processes have seen various elaborate strategies, the first step of template circularization remains less changed. Commonly used method for circular template production is linking a linear oligonucleotide in a head-to-tail way. Two kinds of ligases are used for the head-to-tail ligations. One is double-stranded DNA (dsDNA) ligase, such as T4 DNA ligase and *E. coli.* DNA ligase, the other is ssDNA ligase, such as CircLigase. With the help of a sequence-specific oligonucleotide (splint), template oligonucleotide can be ligated by dsDNA ligase. However, splint should be additionally designed and removed before RCA in some cases^13^,^14^, since remaining linear splint can result in unwanted RCA background^15^. Another ligation method using ssDNA ligase is much simple in a splint-independent way. However, nonspecificity of the ligated substrate DNA limits its application in a mixed nucleic acids reaction system. Moreover, almost all oligonucleotides are solid-phase synthesized from 3′ to 5′ end^16^, therefore, they are deficient in 5′ phosphorate group, which is requisite for DNA ligase activity^17^. Consequently, a phosphorylation process of the linear oligonucleotide is always required before ligation.

In recent years, some groups have noticed the special characteristics of dumbbell probe (DP) employed in RCA, and achieved exciting progress. It was used to detect RNA and transcription factor sensitively by intercalating SYBR Green I into the stem part of DP^18^,^19^. Combined with nickase and G-quadruplex, a DNAzyme method was proposed^20^. It was also used for ATP detection by introducing a third loop into the stem^21^. A target sequence recycled RCA (TR-RCA) has been developed by opening the dumbbell stem with the target sequence^22^. Methyltransferase activity has been analyzed by methylation at the stem part^23^. The cleavage of dumbbell probe was also used in a logic gate operation^24^. However, the production of dumbbell probe by cleavage-ligation of hairpin (HP) has not been proposed to our knowledge.

Here, we presented a circular template production method based on hairpin cleavage-ligation process. Moreover, we explored key factors affecting this method, including the position of RE cleavage sequence in the stem, the primer binding position in the loop, and the usage of different kinds of REs. Furthermore, by employing another round of cleavage-ligation, we realized circle to circle (C2C) amplification to enhance both the sensitivity and specificity of DP RCA obviously. Finally, we proved the feasibility of coreaction system of DP RCA, and provided a more convenient way for DP production and C2C RCA.

## Results and Discussion

### Formation of circular DP by cleavage-ligation of hairpin

**Figure 1:**
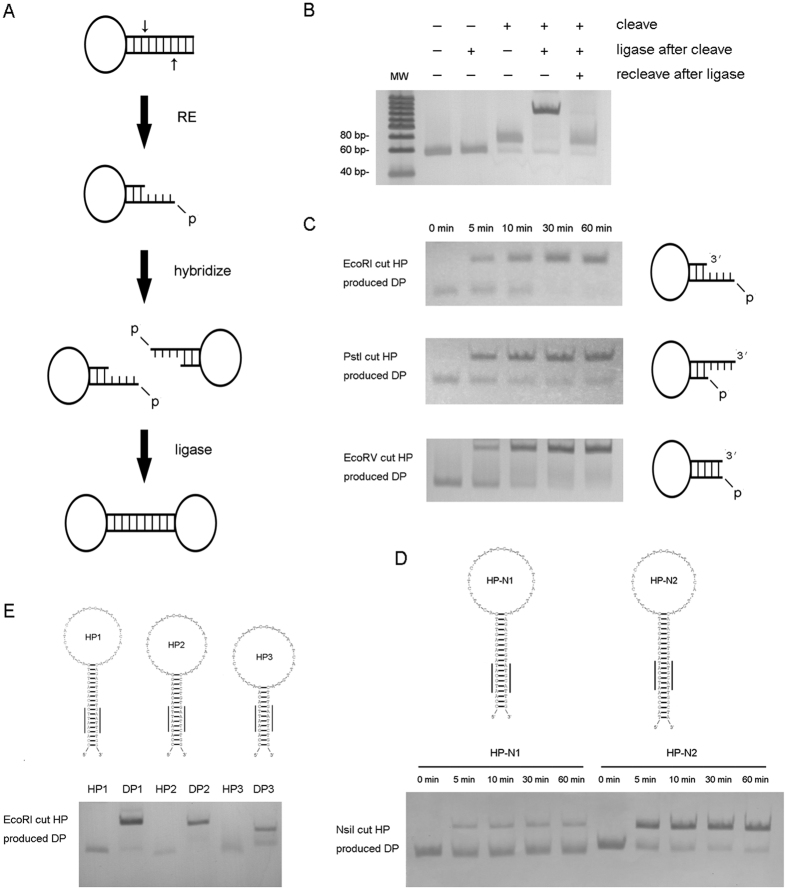
Verification and optimization of hairpin (HP) cleavage-ligation. (**A**) Schematic illustration of dumbbell probe (DP) formation by cleavage-ligation of hairpin. (**B**) DP formation analyzed by PAGE. Only after cleavage, hairpin can be ligated into a band-shifted DP, which can be recleaved into hairpin. (**C**) All of the three kinds of cleaved ends produced by EcoRI, PstI and EcoRV are suitable to form DP efficiently. (**D**) Effects of stem length at the end side of the RE recognition sequence on DP formation. NsiI recognition sequence is underlined. (**E**) Effects of stem length at the loop side of the RE recognition sequence on DP formation. EcoRI recognition sequence is underlined.

The circular template of RCA is traditionally formed by the ligation of a linear template in a head-to-tail way with a requisite 5′ phosphorylation. REs recognize and cleave sequence-specific dsDNA, leaving a 5′ phosphate group at the break site^25^. The phosphate and the sticky ends after cleavage are natural substrates for T4 DNA ligase, as they have been used in molecular cloning. Here, this strategy was introduced into circular template preparation. RE recognition sequence was designed in the stem of a hairpin. After cleavage, hairpin was conveniently self-ligated into dumbbell-shaped circular template i.e. dumbbell probe. Therefore, a hairpin using two simple steps can easily form a DP for RCA (Fig. 1A).

Based on this concept, we designed a hairpin (37 nt loop and 18 bp stem) containing EcoRI recognition sequence (G/AATTC) in the stem, and carried out sequential cleavage and ligation reactions. The products were analyzed by native polyacrylamide gel electrophoresis (PAGE). The results clearly showed a band shift of DP formation by a cleavage-ligation of hairpin, and a reverse band shift by a re-cleavage after ligation (Fig. 1B). To test the RCA activity of the produced DP, the DP was employed in RCA and the products were analyzed by agarose gel electrophoresis, which showed macromolecule products of RCA near sample well, and the DP RCA products can be cleaved into small hairpins. Moreover, a cleaved DP failed to carry out RCA reaction (See Supplementary Fig. S1). These results clearly proved the success in hairpin circularization. Further optimization of reaction time found that ten minutes ligation was sufficient, which was much quicker than the complete cleavage reaction of about 1 hour (Fig. 1), suggested RE cleavage was a rate-limiting step.

As famous tool enzymes, numerous REs can be used for cleavage. Different cleaved ends have been noticed as a factor affecting ligation efficiency in molecular cloning. Therefore, we tested the DP production possibility of all three kinds of RE cleavage ends: 5′ overhang sticky end, 3′ overhang sticky end and blunt end. We chose EcoRI (G/AATTC) to produce a 4 nt long 5′ overhang sticky end, PstI (CTGCA/G) to produce a 4 nt long 3′ overhang sticky end, and EcoRV (GAT/ATC) for blunt end production. Our results showed that both sticky ends and blunt end were suitable for efficient DP production based on hairpin cleavage-ligation, which verified the method to bear a universal application for numerous RE candidates (Fig. 1C).

We found that the position of RE recognition sequence embedded in hairpin stem played key role in cleavage and ligation reactions. The additional base pairs beside recognition sequence at the end of substrate DNA has been found to enhance RE cleavage efficiency, which is called ‘base pairs from end’^26^. However, the optimal length varies according to different REs. Our results showed that a 3 bp-end was sufficient for EcoRI, PstI and EcoRV to complete DP formation. However, for NsiI (ATGCA/T), the 3 bp-end hairpin substrate (HP-N1) produced much less DP than the 6 bp-end hairpin substrate (HP-N2) did (Fig. 1D). In addition, this is the first report to our knowledge to find NsiI cleavage needs a relatively long ‘base pairs from end’. This unexpected result also suggested a feasible new way for RE assay by using DP.

The stem length between RE recognition sequence and the loop might affect both cleavage and ligation, since the disordered loop might interfere with enzyme-substrate interaction. By shortening this part of stem length from 10 bp to 7 and 4 bp (HP1, HP2 and HP3), we found the efficiency of DP formation decreased obviously for 4 bp length by using EcoRI cleavage (Fig. 1E). This might be due to the inhibited hairpin ligation, since the DNA ligase is less efficient when the end distal to join is less than 10 nt^17^,^27^.

### Primer initiation of DP RCA

**Figure 2:**
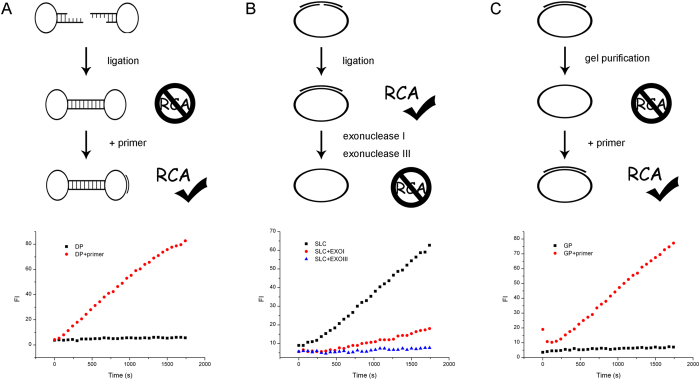
Comparison of the pure circular template background and the primer-initiated RCA. (**A**) RCA fluorescence of hairpin-formed DP with or without primer. (**B**) RCA fluorescence of splint-ligated circular template (SLC) with Exonuclease I or Exonuclease III digestion to purify circular template. (**C**) RCA fluorescence of gel-purified SLC with or without primer. The upper parts are schematic, and the lower parts are fluorescence curves. For comparison, same amounts of circular template of the three methods were used.

A precast circular template is favorable for many RCA techniques^13^,^28^,^29^. The detection kits also prefer a pre-purified circular template. However, the production methods of the head-to-tail ligation always introduce a splint oligonucleotide. Together with unligated template, these linear oligonucleotides can act as primers leaving unwanted detection backgrounds. Therefore, extra laborious purification steps are required to eliminate these linear oligonucleotides, such as Exonuclease I (EXOI) or Exonuclease III (EXOIII) digestion, and denaturing gel purification (GP). Interestingly, we found that the DP formed by cleavage-ligation strategy was free of purification. We compared the two common purification methods (exonuclease digestion and gel purification) of the splint-ligated circular template (SLC) with DP RCA background. Since pure circular template is hard to initiate RCA without primer, our results showed that RCA rates of SLC approaching zero after exonucleases cleavage (Fig. 2B). Through denaturing PAGE and gel cut purification of circular template band, RCA rate can be diminished to baseline and can be also restored by adding a primer (Fig. 2C). However, the formed DP hardly gave a RCA signal, only if a primer oligonucleotide was added (Fig. 2A). Moreover, we found a hairpin oligonucleotide was difficult to initiate DP RCA, which suggested that even there were remaining hairpins from an uncompleted ligation, their contribution to background signal was limited. This might be due to the special duplex ends structure of hairpin. The base-paired stem blocked a free 3′ end and prevented hairpin as a primer to initiate RCA.

**Figure 3:**
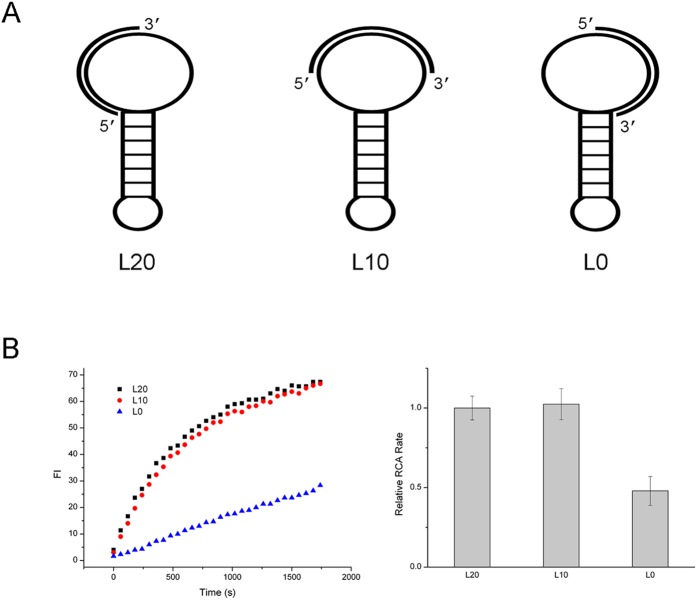
Effects of primer position on DP RCA. (**A**) Schematic diagram of different dumbbell probes. L20: 20 nt from primer’s 3′ end to the stem; L10: 10 nt from primer’s 3′ end to the stem; L0: 0 nt from primer’s 3′ end to the stem. (**B**) The left part is fluorescence time course curve of three dumbbell probes; the right part is relative RCA rates of the three probes. The relative RCA rates were calculated by the slope of fluorescence time course curve normalized by L20.

We also found that the primer binding position designed in template to initiate DP RCA affected RCA efficiency obviously. It has been noticed that the stem part of DP is not suitable for primer initiation, since the stable duplex base pairs prevent primer binding^22^. However, the role of primer position in the loop has not been explored. Therefore, we designed three DPs (12 bp stem and 40 nt loop) with the same primer (20 nt long) complementary to different loop positions of these DPs (Fig. 3A). One was in the middle of the loop (L10) leaving 10 nt gap on each side, the other two binding positions approached stem by 5′ and 3′ end respectively (L20 and L0). We compared the RCA rates of these three DP binding positions and found that in case of L0, RCA efficiency was reduced significantly, while L20 and L10 presented similar amplification rates, which was higher than that of L0 (Fig. 3B). Since DNA polymerase extends from primer’s 3′ end and will encounter the downstream stem, we supposed that the distance from primer’s 3′ end to the stem might be the key point. From L0 to L10 and L20, the distance from primer’s 3′ end to the stem increased gradually (0, 10, 20 nt respectively) which was indeed accord with their RCA efficiency.

### Circle to circle amplification of DP RCA

**Figure 4:**
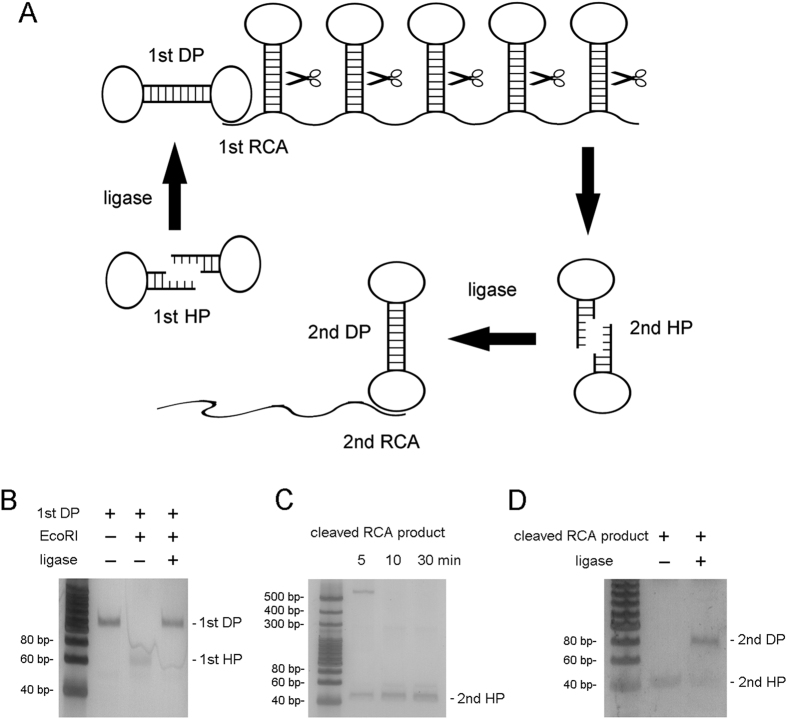
Formation of the 2^nd^ DP from RCA products of the 1^st^ DP. (**A**) Schematic from the 1^st^ HP to the 2^nd^ DP. (**B**) Cleavage and religation of the 1^st^ DP. (**C**) Time course cleavage of the RCA products of the 1^st^ DP. (**D**) The 2^nd^ DP formation from the 2^nd^ HP ligation.

**Figure 5:**
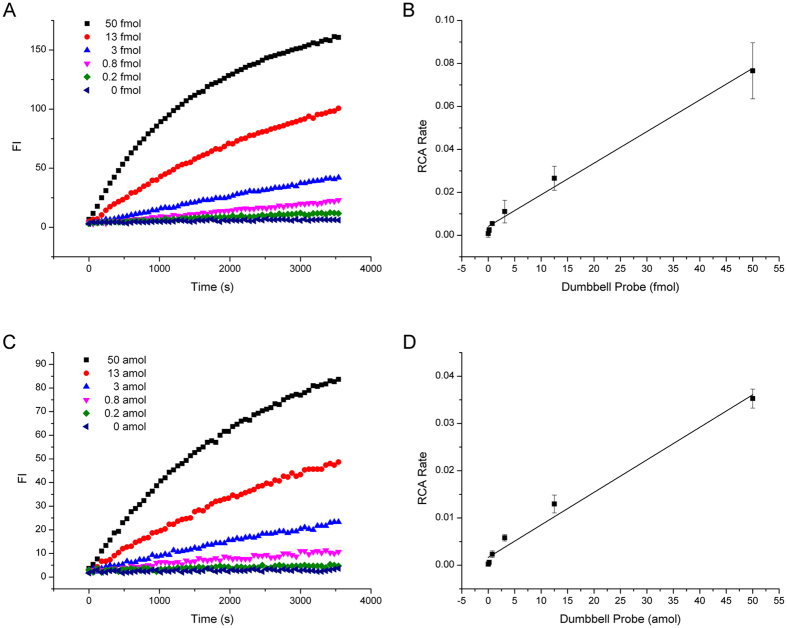
Amplification comparison of the single RCA and the two rounds RCA (C2C RCA). (**A**) Amplification curve of the single RCA by fluorescence detection. (**B**) DP detection linearity by the single RCA. (**C**) Amplification curve of the second round of RCA by fluorescence detection. (**D**) DP detection linearity by the second round of RCA. RCA rates in (**B**,**D**) were calculated by the slope of fluorescence curve in (**A**,**C**) respectively.

DP formed by cleavage-ligation of hairpin is characterized by a RE cleavage site in the stem. This cleavable stem can be passed to the RCA product of DP, which can be easily cut into hairpins (See supplementary Fig. S1). These hairpins in turn can be conveniently ligated into DP again (Fig. 4A). Thus, one original DP can produce hundreds of DPs through RCA amplification. To prove this concept, we named original hairpin as the 1^st^ HP, DP formed by the 1^st^ HP as the 1^st^ DP, hairpin from cleaved RCA products as the 2^nd^ HP, DP formed by the 2^nd^ HP as the 2^nd^ DP. Using gel electrophoresis, the cut site of the 1^st^ DP was verified by being cleaved into the 1^st^ HP and being ligated into DP again (Fig. 4B). Electrophoresis also confirmed that the RCA products of the 1^st^ DP can be cleaved into the 2^nd^ HP gradually for an increasing cleavage time (Fig. 4C), and the 2^nd^ HP was shown to be ligated into the 2^nd^ DP by PAGE analysis (Fig. 4D). Through the amplification of circular template, in comparison with a single round of DP RCA, the detection sensitivity of the second round of DP RCA monitored by microplate reader increased over 1000 times (detection limit from 3.45 fmol to 0.48 amol) with the linear range over two magnitudes (Fig. 5). If another round of amplification is employed, the sensitivity is possible to increase further. Admittedly, during the cleavage of RCA product, the 1^st^ DP can be also cut into the 1^st^ HP, which might hybridize the 2^nd^ HP to interfere with the 2^nd^ DP formation. However, the 1000 folds of RCA amplification produced dominant 2^nd^ HP compared with 1^st^ HP, therefore, this interference can be omitted to some extent.

### Increased specificity by circle to circle DP RCA

**Figure 6:**
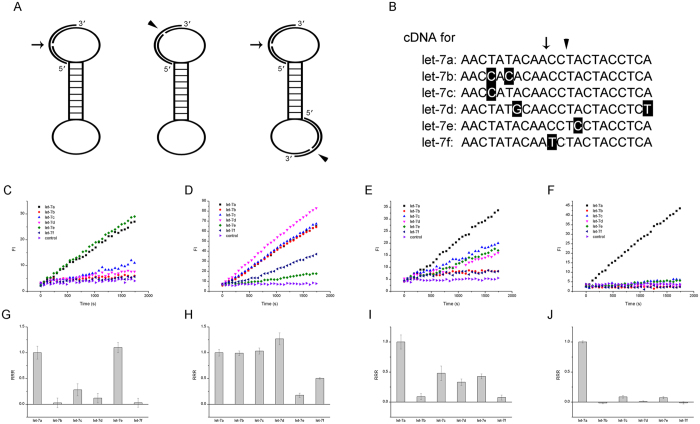
Enhanced specificity by two-ligation design and the 2^nd^ amplification. (**A**) Schematic of the two ligation points: arrow represents the left ligation point and triangle represents the right ligation point. (**B**) Let-7 family sequences alignment marked with ligation points. (**C**~**J**) Family members’ discrimination by RCA. Single left ligation point for discriminating let-7a by fluorescence signal (**C**) and relative RCA rate (**G**); single right ligation point for discrimination by fluorescence signal (**D**) and relative RCA rate (**H**); two ligation points for discrimination by the single RCA fluorescence signal (**E**) and relative RCA rate (**I**); two ligation points for discrimination by the C2C RCA fluorescence signal (**F**) and relative RCA rate (**J**). Relative RCA rates (RRR) in (**G**~**J**) were calculated by the slope of fluorescence curve in (**C**~**F**).

Primer initiation for a precast circular template is convenient for common sequence detection; however, it is still difficult to discriminate highly similar sequences. Let-7 family is a conserved miRNA family which bears high sequence similarity^30^. As a model for high specific assay, we designed several DP detection probes for discriminating let-7a from other five similar members (let-7b~7f). The loop of DP complementary to let-7a cDNA (clet-7a) was circularized and ligated by T4 DNA ligase. Two ligation points in the middle of clet-7a sequence were designed. One was located between A and C, which we called the left ligation point (the left diagram of Fig. 6A,B), the other was between C and T, called the right ligation point (the middle diagram of Fig. 6A,B). We compared the detection specificity of these two ligation sites. By using the left ligation point, we can only discriminate clet-7a from 7b, 7c, 7d and 7f, but not 7e (Fig. 6C,G). When we used the right ligation point, clet-7a can be only discriminated from 7e and 7f, but not 7b, 7c and 7d (Fig. 6D,H). The results were reasonable when we noticed the proximity of mismatch position to the ligation point. The mismatches of clet-7b, 7c and 7d were near the left ligation point; therefore they can be discriminated by left ligation point but not right ligation point. The mismatch of clet-7e was close to the right ligation point which therefore can discriminate it easily (Fig. 6B). For the purpose of distinguishing all these family members simultaneously, we embedded both of these two ligation points at each loop in one DP (the right diagram of Fig. 6A). Thus, the DP can be circularized only in the case of linking both of these two kinds of ligation points. Our results showed that this strategy achieved effective discrimination for all the tested members (Fig. 6E,I). Furthermore, after the first amplification of DP, we conducted the second amplification of the 2^nd^ DP through C2C process, and acquired even higher specificity (Fig. 6F,J). These results suggested that the two-ligation design and two rounds of amplification of DP efficiently enhanced its detection specificity.

### Coreaction feasibility of multiple reactions

C2C amplification has been introduced into common RCA and achieved high sensitivity in previous reports^31^,^32^. However, the steps of C2C RCA are laborious, since each cycle includes adding splint, cleavage, ligation and polymerization. Therefore, a more convenient way for C2C is extremely attracting. For DP RCA combined with C2C, the cleavage and ligation are naturally merged into the stem part in circular template free of designing additional splint oligonucleotide. Here, we further proved the feasibility of DP formation by coreaction of multiple reactions to acquire more convenience.

**Figure 7:**
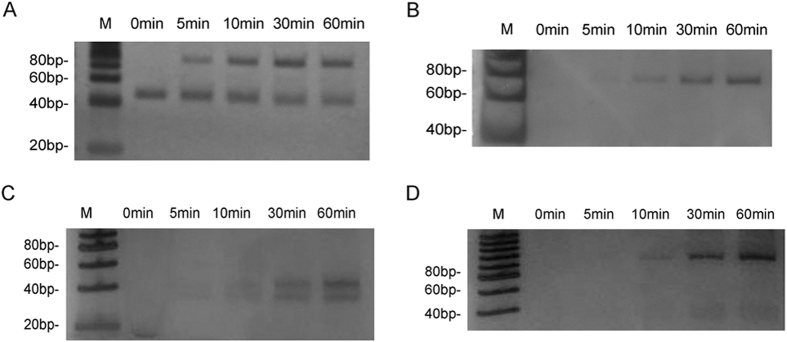
Coreaction of multiple reactions. (**A**) The 1^st^ DP formation by coreaction of hairpin cleavage and ligation. (**B**) The 2^nd^ DP formation by coreaction of cleavage and ligation after the 1^st^ DP RCA. (**C**) Coreaction of the 1^st^ DP polymerization and cleavage. (**D**) The 2^nd^ DP formation by ligation from the 2^nd^ HP in (**C**).

For the 1^st^ DP formation, we conducted the cleavage and ligation of hairpins at the same time, and electrophoresis showed that DP band accumulated gradually with the decrease of hairpin band (Fig. 7A). This can be explained by the instability of the cleaved stem end, which was only 3 base-paired, therefore, was instable in reaction temperature and dispersed instantly. Then, the following ligation between cleaved hairpins produced the 1^st^ DP.

For the 2^nd^ DP formation, the RCA product of the 1^st^ DP was cleaved and ligated simultaneously. Results verified the enriched 2^nd^ DP band by PAGE analysis (Fig. 7B). The cleavage of RCA macromolecule product quickly dispersed large amounts of cleaved 2^nd^ HPs before ligase can act. Consequently, after dimerization, hairpins were ligated into the 2^nd^ DP. Another reason for DP accumulation was that we used much higher ligase activity than RE activity. Therefore, DP was preferred to be ligated rather than to be cleaved.

Another coreaction effort was the combination of polymerization and cleavage reactions, since these two reactions were rate-limiting steps which were much slower than ligation process. However, except RCA product, the dumbbell circular template can be also digested by REs, and a cleaved circular template fails to carry out RCA reaction anymore. Therefore, we introduced nucleotide analog to protect template from RE cleavage. An optimum analog should resist RE cleavage without interfering with polymerization reaction of RCA. From a selecting study, we found phosphorothioate (PS) modification at A5pG6 position can strongly suppress PstI activity, and PS had little effects on RCA efficiency^33^,^34^. Consequently, we used DP with a PS-modified PstI hexamer in the stem to conduct this coreaction. Results showed a successful accumulation of the 2^nd^ HP during the coreaction process (Fig. 7C), and after a quick ligation reaction (10 minutes), the 2^nd^ HP turned into the 2^nd^ DP conveniently (Fig. 7D).

In summary, we presented a cleavage-ligation strategy to produce circular dumbbell probe. It has the advantages of phosphorylation-free, splint-free and purification-free. The position of RE recognition sequence in the stem affected DP formation obviously. Both sticky ends and blunt end cleaved by REs were efficient for DP formation. The primer position in the loop also affected DP RCA efficiency obviously. By introducing C2C into DP RCA, the amplification rate increased about 1000 times. Combined with a two-ligation strategy, DP C2C RCA achieved extremely high specificity for let-7 family discrimination. The coreaction of multiple reactions provided more convenient operations.

## Methods

### Oligonucleotides and reagents

Oligonucleotides at HPLC grade were purchased from Sangon Biotech Co. Ltd. (Shanghai, China). The concentration was determined by the absorption coefficiency of each sample. Their sequences were listed in Supplementary Table S1, and the oligonucleotides for forming the circular templates had a phosphoric acid group at the 5′-terminal. Phi29 DNA polymerase and NsiI restriction endonuclease were purchased from NEB (Ipswith, MA). SYBR Green II was from Invitrogen (Waltham, MA). T4 DNA Ligase and restriction endonucleases EcoRI, EcoRV and PstI were from TaKaRa Biotechnology Co. Ltd. (Dalian, China).

### SLC preparation

For splint-mediated ligation, the 5′-end of the circular template was phosphorylated. The 5′- and 3′-portions of the circular template (1 μM) were hybridized with 1 μM of splint oligonucleotide in a head-to-tail fashion, and were covalently linked by T4 DNA ligase (175 U) at 16 °C for 60 min. Ligation buffer contains 50 mM Tris pH 8.0, 10 mM MgCl_2_, 5 mM DTT, and 0.1 mM ATP in 10 μl reaction system.

### DP preparation through RE cleavage-ligation

For hairpin-mediated ligation, hairpin of 1 pmol was cleaved by RE. The cleavage reaction was carried out in 10 μl reaction buffer containing 50 mM Tris pH 7.5, 10 mM MgCl_2_, 1 mM DTT, and 100 mM NaCl. The cleavage was initiated by adding 5 U REs (EcoRI, EcoRV, PstI or NsiI). After incubation at 37 °C for 30 min, the cleavage was stopped by heating at 90 °C for 10 min. After cleavage, by adding 1 nmol ATP and 175 U T4 DNA ligase, ligation reaction was conducted at 37 °C for 10 min.

### Exonuclease digestion and gel purification

After SLC preparation described above, the 1 pmol splint-ligated circular template was digested by Exonuclease I 5 U or Exonuclease III 5 U respectively in 10 μl reaction system at 37 °C for 30 minutes. After digestion, the exonucleases were deactivated at 80 °C for 15 minutes. 0.1 pmol undigested and digested SLC were applied for RCA respectively. For gel purification process, SLC was verified and separated by 9% denatured PAGE (7 M urea) and silver staining as described^35^. The circular template band was cut after electrophoresis. The gel was crushed, and the circular template was extracted by H_2_O and centrifugation. Concentration of the purified circular template was determined by ultraviolet at 260 nm. 0.1 pmol gel-purified SLC was applied with or without primer for RCA respectively. RCA procedure was described below.

### Rolling circle amplification

0.1~1 pmol ligated circular template with or without primer were added into RCA reaction solution which was composed of 50 mM Tris pH 7.5, 10 mM MgCl_2_, 10 mM (NH_4_)_2_SO_4_, 4 mM DTT, SYBR Green II (1:10000), and phi29 DNA polymerase 3 U in 100 μl solution. Fluorescence signals were recorded on Microplate Reader (Infinite M200, Tecan, USA) with excitation wavelength at 480 nm and emission wavelength at 524 nm. The fluorescence emission was monitored for over ~20 min at 37 °C.

## Additional Information

**How to cite this article**: Wei, H. *et al*. Production of dumbbell probe through hairpin cleavage-ligation and increasing RCA sensitivity and specificity by circle to circle amplification. *Sci. Rep.* **6**, 29229; doi: 10.1038/srep29229 (2016).

## Supplementary Material

Supplementary Information
