# Supplementary material for: Production of dumbbell probe through hairpin cleavage-ligation and increasing RCA sensitivity and specificity by circle to circle amplification
Source: Sci Rep. 2016 Jul 7;6:29229. doi: 10.1038/srep29229 (PMC4935871; doi:10.1038/srep29229)
Supplement: Supplementary Information [file srep29229-s1.doc]

**Production of dumbbell probe through hairpin cleavage-ligation and increasing RCA sensitivity and specificity by circle to circle amplification**

Hua Wei^1, 2^, Suming Tang^2^, Tianyu Hu^2^, Guojie Zhao^2, *^, and Yifu Guan^2, *^

^1^Animal Science and Veterinary Medicine College, Shenyang Agricultural University, #120 Dongling Road, Shenyang, Liaoning, 110866, China

^2^Department of Biochemistry and Molecular Biology, China Medical University, #77 Puhe Road, Shenyang, Liaoning, 110122, China

^*^Correspondance and requests for materials should be addressed to G.Z. ([gjzhao@mail.cmu.edu.cn](mailto:gjzhao@mail.cmu.edu.cn)); Y.G. ([yfguan@mail.cmu.edu.cn](mailto:yfguan@mail.cmu.edu.cn))

**ABSTRAT**

**Dumbbell probe (DP) attracts increasing interests in rolling circle amplification (RCA). A universal DP production method through cleavage-ligation of hairpin was proposed and optimized. The production is characterized by restriction endonuclease (RE)-induced cleavage ends ligation. It has the advantage of phosphorylation-free, splint-free and purification-free. To optimize designing, we found that the position of RE cleavage sequence in the stem and the primer position in the loop affected the formation and amplification of DP obviously. Both sticky and blunt ends cleaved by RE produce DP efficiently. Moreover, we introduced this DP into circle to circle (C2C) RCA based on the same cleavage-ligation principle, and acquired high sensitivity. By combining a two-ligation design and the C2C strategy, specificity for detecting let-7 family members was increased extremely. Furthermore, coreaction of different steps facilitated convenient formation and amplification process of DP.**

Supplementary information

**Figure:**


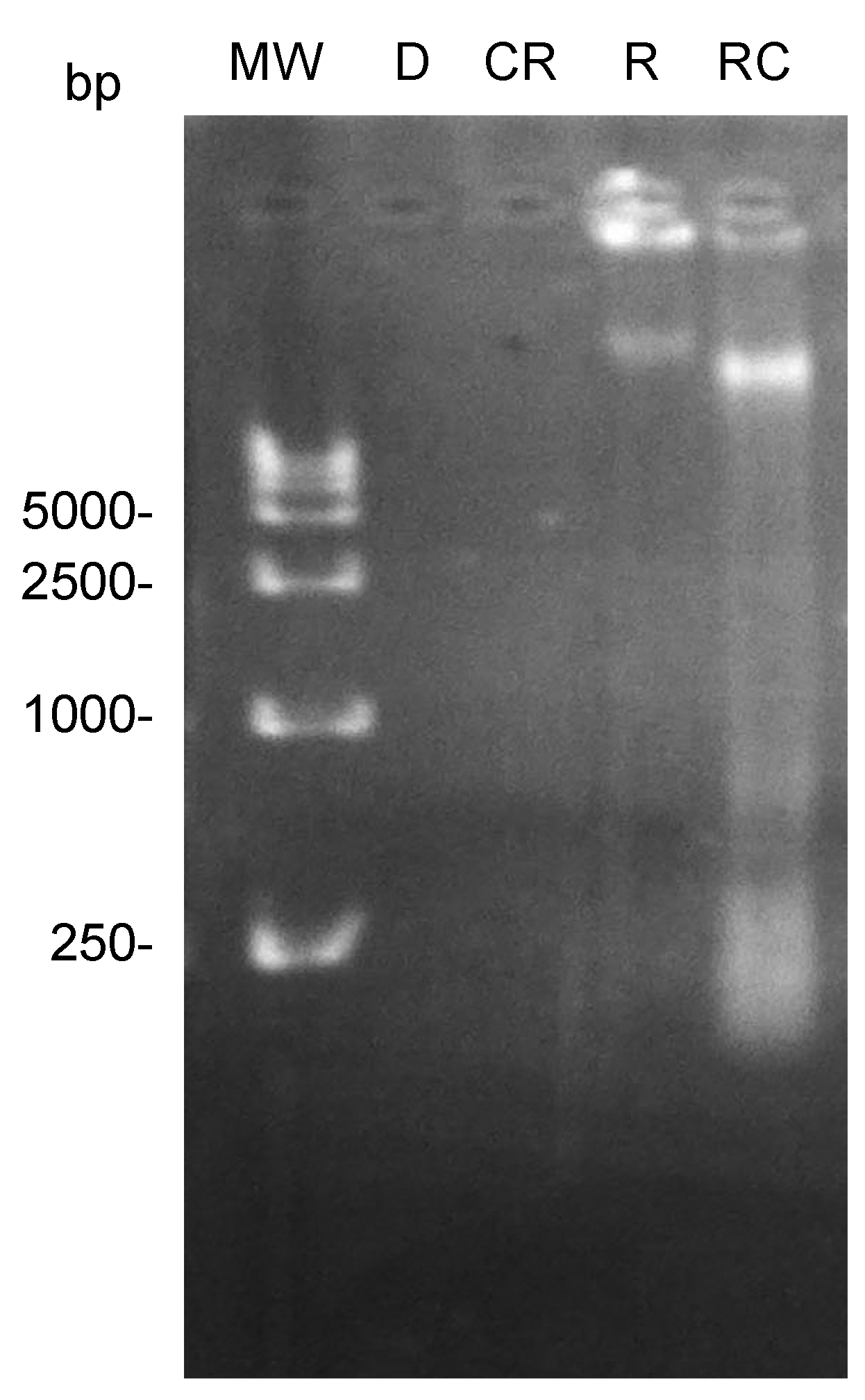


**Figure S1** | **RCA analysis of DP by agarose gel electrophoresis.** Lane D: DP without phi29 DNA polymerase; Lane CR: cleaved DP by RE with phi29 DNA polymerase; Lane R: DP with phi29 DNA polymerase; Lane RC: DP RCA product cleaved by RE.

**Table:**

**Table S1** | **Oligonucleotide sequences in this study**

| Name | Sequence* |
| --- | --- |
| For concept proving | |
| HP-EcoRI | 5'-*CCAGAATTCACAGAGTCT-*TTATTAATCTTTGGTTGAGGTAGTAGGTTGTATAGTT-*AGACTCTGTGAATTCTGG*-3' |
| HP-EcoRV | 5'-*CCAGATATCACAGAGTCT-*TTATTAATCTTTGGTTGAGGTAGTAGGTTGTATAGTT-*AGACTCTGTGATATCTGG*-3' |
| HP-PstI | 5'-*CCACTGCAGACAGAGTCTG-*ACTTTCTACTTATCGATTAATCATTGGA-*CAGACTCTGTCTGCAGTGG*-3' |
| HP-P1 | 5'-GTCCAATGATTAATC-3' |
| For base pairs from end | |
| HP-N1 | 5'-*CCAATGCATACAGAGTCTG-*ACTTTCTACTTATCGATTAATCATTGGA-*C*  *AGACTCTGTATGCATTGG*-3' |
| HP-N2 | 5'-*TAGCCAATGCATACAGAGTCTG-*ACTTTCTACTTATCGATTAATCATTGGA-*CAGACTCTGTATGCATTGGCTA*-3' |
| HP-P1 | 5'-GTCCAATGATTAATC-3' |
| For intervals between loop and recognition sequence | |
| HP1 | 5'-*CCAGAATTCACAGAGTCTG-*ACTTTCTACTTATCGATTAATCATTGGA  *-CAGACTCTGTGAATTCTGG*-3' |
| HP2 | 5'-*CCAGAATTCACAGAGG-*ACTTTCTACTTATCGATTAATCATTGGA-*CCT*  *CTGTGAATTCTGG*-3' |
| HP3 | 5'-*CCAGAATTCACAG-*ACTTTCTACTTATCGATTAATCATTGGA-*CTG*  *TGAATTCTGG*-3' |
| HP-P1 | 5'-GTCCAATGATTAATC-3' |
| For primer initiation | |
| SLC | 5'-TGAGCACTAGTTATCCCATCTAGAACACGCAATGTTCTAGATGGTCTACATCATTGGAC-3' |
| SLC-P | 5'-CTAGTGCTCAGTCCAATGAT-3' |
| For primer position | |
| L10 | 5'-TGAGCACTAGTTATCGATTA-*CCATCTAGAACA-*CGCAA-*TGTTCTAGATGG-*GAGTTTCTACATCATTGGAC-3' |
| L20 | 5'-TGAGCACTAG-*CCATCTAGAACA-*CGCAA-*TGTTCTAGATGG-*GACTTTCTACTTATCGATTAATCATTGGAC-3' |
| L0 | 5'-TGAGCACTAGTTATCGATTAGAGTTTCTTC-*CCATCTAGAACA-*CGCAA-*TGTTCTAGATGG-*ATCATTGGAC-3' |
| SLC-P | 5'-CTAGTGCTCAGTCCAATGAT-3' |
| For sensitivity | |
| HP-EcoRIb | 5'-*CCAGAATTCACAGAGTCT*-CACTTTCTACTTATCTTCAATCGCTTTTCA  *-AGACTCTGTGAATTCTGG*-3' |
| HP-EcoRIb-P | 5'-TGAAAAGCGATTGAA-3' |
| HP-EcoRIb-P2 | 5'-CACTTTCTACTTATC-3' |
| For specificity | |
| DP-let-eco-L | 5'-TTGTATAGTT-*AGACTCTGTGAATTCACAGAGTCT*-TTATTAATCTTTGGT-TGAGGTAGTAGG-TTGTATAGTT-*AGACTCTGTGAATTCACAGAGTCT*-TTATTAATCTTTGGT-TGAGGTAGTAGG-3' |
| DP-let-eco-R | 5'-GGTTGTATAGTT-*AGACTCTGTGAATTCACAGAGTCT*-TTATTAATCTTTGGT-TGAGGTAGTAGG-TTGTATAGTT-*AGACTCTGTGAATTCACAGAGTCT*-TTATTAATCTTTGGT-TGAGGTAGTA-3' |
| DP-let-ecoI-LR1 | 5'-TTGTATAGTT-*AGACTCTGTGAATTCCCTATGAAG*-TTATTAATCTTTGGT-TGAGGTAGTA-3' |
| DP-let-ecorI-LR2 | 5'-GGTTGTATAGTT-*CTTCATAGGGAATTCACAGAGTCT*-TTATTAATATTTGGT-TGAGGTAGTAGG-3' |
| clet7a | 5'-AACTATACAACCTACTACCTCA-3' |
| clet-7b | 5'-AACCACACAACCTACTACCTCA-3' |
| clet-7c | 5'-AACCATACAACCTACTACCTCA-3' |
| clet-7d | 5'-AACTATGCAACCTACTACCTCT-3' |
| clet-7e | 5'-AACTATACAACCTCCTACCTCA-3' |
| clet-7f | 5'-AACTATACAATCTACTACCTCA-3' |
| clet7a-p2 | 5'-TTATTAATCTTTGGT-3' |
| For coreaction | |
| DP-let-pst-s | 5'-TTGTATAGTT-*AGACTCTGTCTGCA*GACAGAGTCT-*TTATTAATCTTTGGTTGAGGTAGTAGGTTGTATAGTT-*AGACTCTGTCTGCA*GACAGAGTCT-*TTATTAATCTTTGGTTGAGGTAGTAGG-3' |

*The RE recognition sequences are underlined. The stem parts are in italic type. Asterisk represents the phosphorothioate-modified site of RE recognition sequence.
